# Widespread occurrence of pink salmon (*Oncorhynchus gorbuscha*) throughout Greenland coastal waters

**DOI:** 10.1111/jfb.14318

**Published:** 2020-03-24

**Authors:** Julius Nielsen, Aqqalu Rosing‐Asvid, Lorenz Meire, Rasmus Nygaard

**Affiliations:** ^1^ Department of Fish and Shellfish Greenland Institute of Natural Resources Nuuk Greenland; ^2^ Department of Birds and Mammals Greenland Institute of Natural Resources Nuuk Greenland; ^3^ Greenland Climate Research Centre Nuuk Greenland; ^4^ Department of Estuarine and Delta Systems Royal Netherlands Institute of Sea Research, Utrecht University Yerseke Netherlands

**Keywords:** Arctic, distribution, invasive, non‐indigenous, river, salmonid

## Abstract

Using social media, the Greenland Institute of Natural Resources collected data on the occurrence of pink salmon (*Oncorhynchus gorbuscha*) in 2019. Eighty‐four pink salmon were reported from 22 locations across Greenland. This comprised 76 specimens from 2019 and 8 specimens from 2013 to 2018. Of these, 12 were caught in fresh water, and a single pink salmon was from the bottom of the Nuuk Fjord near the Kapisillit River – the only known river in Greenland where the Atlantic salmon (*Salmo salar*) spawn. It is unknown if pink salmon have reproduced in Greenland waters.

1

The migratory pink salmon (*Oncorhynchus gorbuscha*) has a large native range with breeding populations across the North Pacific, including parts of the Bering Sea and the Arctic Ocean (Page & Burr, 1991). Spawning generally occurs in the lowermost parts of rivers and streams and with a typical 2‐year anadromous life cycle; odd‐year populations of pink salmon are reproductively separated from populations breeding in even years (Heard, 1991). Since the 1950s, pink salmon has repeatedly been introduced adjacent to the Atlantic region through Russian stocking programmes on the Kola Peninsula in the White Sea (Sandlund *et al*., 2019; Zubchenko *et al*., 2010). Consequently, non‐indigenous pink salmon have been reported for decades occasionally across the northern North Atlantic, including Greenland, where they were first observed in 1969 (Møller *et al*., 2010; Sandlund *et al*., 2019). The Russian stocking programmes ceased in the early 2000s; yet in 2017, a so‐called invasion of pink salmon was reported across northern Europe, evidencing that pink salmon have successfully established and are reproducing in the northeast Atlantic region (Millane *et al*., 2019; Mo *et al*., 2018). The pink salmon “invasion” has been most noticeable in Norway, where they have been reported in hundreds of rivers causing great concerns in terms of the potential associated negative effects on indigenous salmonids (Gjelland & Sandlund, 2012; Hindar *et al*., 2020; Mo *et al*., 2018; Sandlund *et al*., 2019). The pink salmon is currently considered an invasive species throughout northern Europe and its distribution is expected to increase in coming years.

In 2017, the Greenland Institute of Natural Resources (GINR) received five reports of five pink salmon in the coastal waters of Greenland (GINR, unpublished data; ICES, 2018). In August 2019, GINR was contacted by two fishermen who had caught multiple pink salmon near the settlement of Tasiilaq in southeast and near Nuuk in southwest Greenland, respectively. The following week, GINR initiated an awareness campaign on Facebook – the most used social media in Greenland where 64% of the population has a profile (Nordlund, 2016). The public was encouraged to report observations of pink salmon. Within the following 4 weeks, reports of pink salmon with associated capture location, estimated length and photograph came from across all inhabited areas of both east and west Greenland (except the northernmost settlements in west Greenland, not shown in Figure 1). In total, 83 pink salmon were reported from 22 different locations across Greenland (Figure 1). Of these, one report was from 2013, when a fisherman had caught one pink salmon using a fishing rod in a river near Upernavik. Another report was from 2015, three reports were from 2017 and four were from 2018. The remaining 74 reports were from the summer or early autumn of 2019 (July–September). Of all the reported pink salmon, 12 were caught in three different rivers (see freshwater locations in Figure 1). The remaining fish were from coastal waters often near river outlets, where pink salmon had been caught as a by‐catch in gillnets targeting Arctic char (*Salvelinus alpinus*) or Atlantic salmon (*Salmo salar*). No accurate length measurements were obtained from the survey, but from fishermen's estimates as well as available photographs, it is estimated that all specimens measured approximately 50–60 cm in total length. At least two females were reported to have ripe eggs and three males to have freely flowing sperm (in Nuuk, Uummannaq and Qaqortoq areas from mid‐August to mid‐September, Figure 1). One observation of a pink salmon male was from the bottom of the Nuuk Fjord only 500 m away from the Kapisillit River outlet (Figure 1), which is the only known river in Greenland with a spawning population of Atlantic salmon (Hedeholm *et al*., 2018). The data collection of this study did not require the use of experimental animals, and all fishing activities, from where data have been acquired, were conducted in accordance with national laws and regulations.

**Figure 1:**
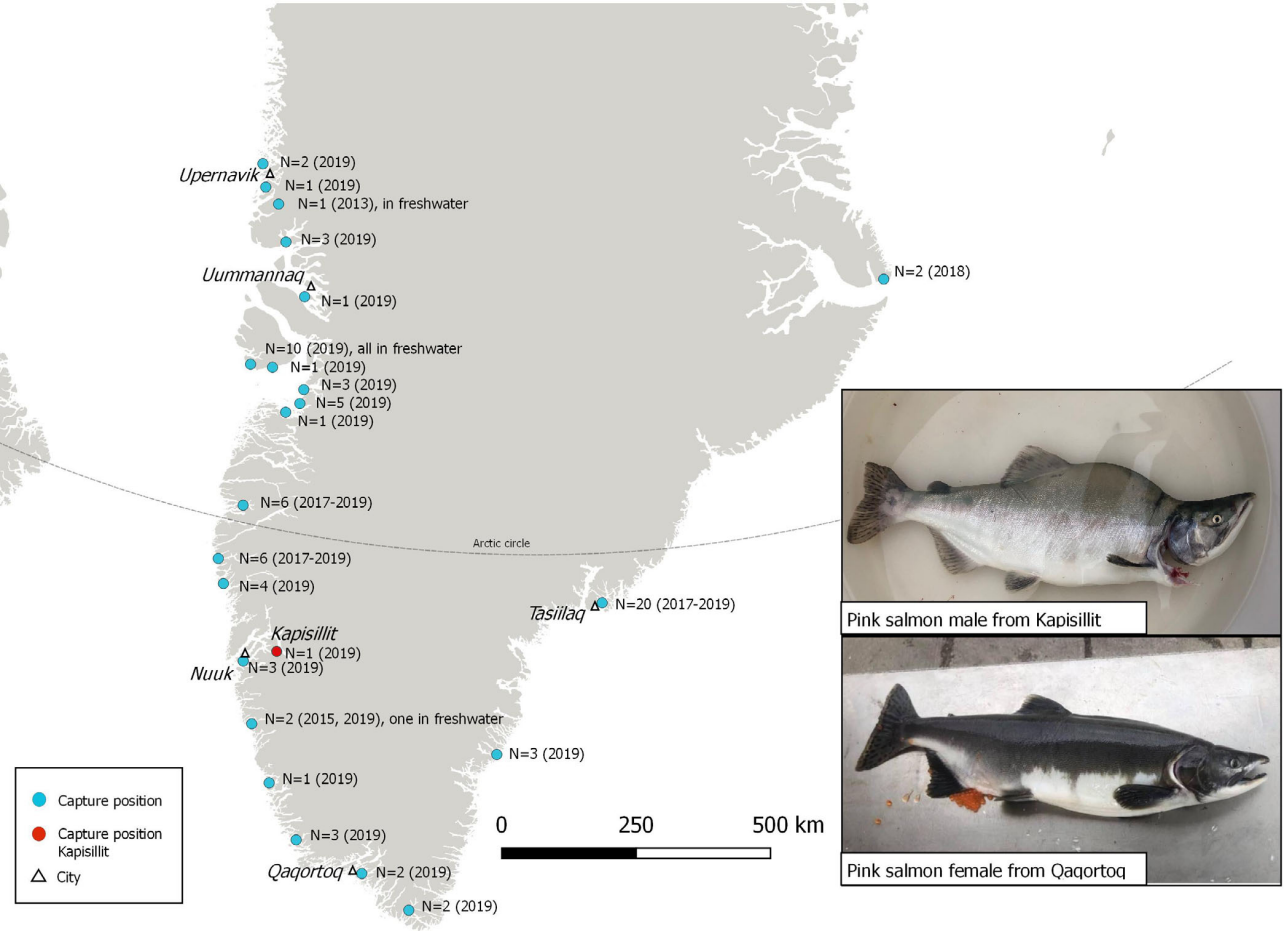
Capture locations of pink salmon in Greenland. Blue dots represent capture positions of pink salmon. The Kapisillit capture location is marked with a red dot. The number, *N*, and year are shown for each dot. Notice the three freshwater capture positions. Larger cities are marked with a triangle. Photo credit: Heidi Lindholm and Jens Sikemsen

It is fair to consider that pink salmon are present in Greenland waters in high numbers, but whether the abundance in 2019 is higher than that in 2017 and 2018 cannot be evaluated from the available data. Nonetheless, in 2017 pink salmon were found in unprecedented numbers in multiple rivers in, for example, Norway (mainland and Svalbard), Denmark, Ireland and Scotland (ICES, 2018). The unprecedented high number of reports of pink salmon in 2019 in both the west and east coasts of Greenland coincides with the reported increased occurrence in northwestern European rivers in both 2017 and 2019, and indicates that pink salmon have the potential to successfully expand their range in the wider Atlantic region, including the Arctic. Besides the strong 2017 generation in northern Europe, the high presence of pink salmon in Greenland in 2019 can possibly be linked to higher sea surface temperatures in the region (Timmermans & Ladd, 2019). Increasing sea temperatures are attributed to an increased abundance of pink salmon in Atlantic parts of the Arctic (Hindar *et al*., 2020).

The establishment of pink salmon in Atlantic rivers is associated with the possibility of negative effects on local salmonids because of potential competition, infectious diseases and parasites, as presented in Hindar *et al*. (2020). In Greenland, Arctic char and Atlantic salmon are the only two native salmonids. The Arctic char is highly abundant and widespread in both rivers and lakes on the west and east coasts of Greenland. The Atlantic salmon is known to reproduce only from a single population in the Kapisillit River in the bottom of the Nuuk Fjord, southwest Greenland (Figure 1). Higher temperature in the Kapisillit River (compared to other rivers in Greenland) is likely the main factor allowing Atlantic salmon to reproduce in the lower parts of this river system (Hedeholm *et al*., 2018). Therefore, the presence of pink salmon near the outlet of this river is causing concern, as it seems a very likely location in Greenland for pink salmon to establish and spawn successfully. The Atlantic salmon population in the Kapisillit River is listed as “Vulnerable” on the Greenland Red List (Boertmann & Bay, 2018), and the population has recently been found to be declining, supposedly because of unregulated fishing activities in the river and nearby estuary (Hedeholm *et al*., 2018). The presence of pink salmon in Greenland should be considered a new potential threat towards Atlantic salmon, as well as a potential threat towards Arctic char elsewhere in Greenland.

## AUTHOR CONTRIBUTIONS

J.N. coordinated this work and prepared the manuscript. All authors equally conceived the idea; contributed to scientific discussions; and revised, reviewed and finally approved the work.
